# Extracts from the Report of a Committee of the Boston Society for Medical Improvement, on the Alleged Dangers Which Accompany the Inhalation of Vapor of Sulphuric Ether

**Published:** 1861-12

**Authors:** 


### EDITORIAL DEPARTMENT.

### EXTRACTS FROM THE REPORT OF A COMMITTEE OF THE BOSTON SOCIETY FOR MEDICAL IMPROVEMENT, ON THE ALLEGED DANGERS WHICH ACCOMPANY THE INHALATION OF VAPOR OF SULPHURIC ETHER.

Any one who has observed the course of events, especially the tone of journals and the published statements of late surgical writers, as Erichsen, Druitt, Hamilton and others, must have noticed a diminishing confidence in the safety of chloroform and an increasing willingness to allow the greater security of ether. Various influences have, however, prevented the disuse of the former, even by many of those in whose hands accidents have occurred, and it still remains the anæsthetic most in vogue. When the subject of chloroform first came under discussion, its dangers were commented upon, and even then freely acknowledged. It had not been two months introduced, when "a well-developed girl of fifteen" died from its administration for the evulsion of a toe-nail, "the process of inhalation, operation and death not having occupied more than two minutes." Since that time, deaths from its use have repeatedly occurred. On the other hand, fatal results from ether, although still figuring in the statistics of mortality from anæsthetics, are everywhere admitted to be very infrequent. Indeed, the opinion has been expressed by various authorities, both in America and Europe, that a death really attributable to the inhalation of sulphuric ether is yet to be reported. The correctness of this opinion has, however, been repeatedly denied, and the strong conviction of the absolute safety of this agent, which exists in some localities in this country, is thought to have its foundation rather in the desire that the fact might be established than in the proof that it was so. Of course no one intends to say that a person cannot be killed by ether. The inhalation of its vapor, without a sufficient admixture of oxygen, destroys life by asphyxia. This may happen, and unfortunately has happened, but such an event cannot be laid to the anæsthetic, since, in such a case, it is the method of administration, and in no sense the ether, which causes the fatal result.

It is the purpose of this report to solve the doubt just implied with regard to the absolute safety of sulphuric ether, and to investigate the dangers of its use as compared with chloroform. In pursuance of this object, therefore, we propose, in the first place, to consider what conditions and precautions are necessary in bringing about a state of insensibility by its use, and what phenomena of etherization have an apparent or real danger.

I.—The safe inhalation of ether requires proper attention—1st, to the quality of the article used; 2d, to the method of administration; 3d, to the symptoms which present themselves while the patient is under its influence.

1st. *Quality of Ether.*—Ether for inhalation should be of unquestionable purity. A large amount of inferior ether is sold which cannot readily be distinguished from that which is pure, except by its effects, although an expert, familiar with its properties, may infer something from the odor and other sansible qualities. The inferiority may be due to oxydation from bad corking, the presence of alcohol, sulphurous acid which has not been removed by thorough washing, and volatile oils. Either of these impurities may give rise to a tedious and imperfect inhalation, and the latter of them, by irritating the bronchial mucous membrane, to such coughing, struggles and resistance that the patient is finally etherized in a distressing and unsatisfactory manner. Accidents of this kind lead to a disparagement of the value and practical usefulness of ether. It is therefore an advantage for the surgeon to procure his own ether, or to use from an "original package," of the character of which he has already assured himself.

There are two brands of ether in common use in this country, viz: that manufactured by Powers & Weightman, of Philadelphia, and that by Dr. Squibb, of Brooklyn, N. Y. These are uniformly of excellent quality. The latter is remarkably anhydrous, but possesses an odor more harsh, disagreeable, and intensely ether-like than the former, and, in the opinion of those who heve used it extensively, produces more choking during inhalation. This may be remedied to a certain extent by moistening the sponge from which it is given in water, enough of which will perhaps be taken up by the ether to diminish its unpleasant effects.

Ether may be made purer by simple agitation in lime-water, allowing the water to settle, and then decanting; and this washing is, practically, and for general application, as good a method of purification as can be adopted without re-distillation.

2d. *Method of Administration.*—Ether should never be given from any inhaling apparatus. The best medium of its administration is a bell-shaped sponge, large enough to cover in the nose, mouth and chin; but it s difficult to find one of sufficient size and close enough in texture, or without such numerous apertures at the root as to admit too freely the atmospheric air. A sponge of this sort, moreover, being as expensive as rare, is seldom used outside of hospitals. A stiff towel, properly folded, may be substituted, and has the advantage of being always at hand; as it may be left behind, the surgeon does not carry away with him the annoying odor of an impregnated sponge. It is desirable that the towel should be a new one, and of pretty good size. It is to be taken just as it comes from the laundry, and not unfolded further than to display it in the dimensions of about ten inches by five; by folding down two of the corners in such a way that they shall lap over each other a little, and securing them by stout pins, a cone will be made which fits the face admirably. The thick layers of towelling will hold sufficient ether, and its texture prevent a too free dilution of the anæsthetic by the atmospheric air, provided the apex and seam of the cone are carefully and tightly closed, either by pins or the fingers. As the cone becomes collapsed by saturation, it should from time to time be opened, and kept in shape by distending it with the hand. Unless these details are attended to, and especially the closure of the apex of the cone, the induction of anæsthesia will be uncertain and protracted. In anything so porous as a towel or sponge, the difficulty is to exclude enough air; for while its adequate admission to the lungs during etherization is essential to the life of the patient, its too free entrance not only delays anæsthesia, but induces a condition of excitement, both mental and physical. The importance of excluding the air, as above stated, is a point not generally appreciated, but the necessity of it has long been known to those most accustomed to the use of ether, as shown by the "chemise" with which, in hospital practice, a too porous sponge is often covered to expedite the etherization of a rebellious patient.

Ether should be poured lavishly on the towel or sponge, an ounce or two at a time, especially at the commencement of inhalation. Although it may be wasted, too much, so far as safety is concerned, cannot be used. A small quantity poured on hesitatingly and timidly, as is sometimes done, has the same effect as a too free dilution of the vapor with air, producing simply intoxication and its accompanying excitement without anæsthesia; whereas a large amount, though the cough and choking sensation which the greater volume of vapor produces may cause the patient to resist and struggle, is certain to bring about a satisfactory condition of insensibility.

3d. *Phenomena of Etherization.*—A strong, full-blooded man is pretty sure to resist the approaches of anæsthesia under any circumstances. This may sometimes be overcome by warning him before hand of such a possibility, and inducing him to resolve not to struggle; the last impression on his mind influences him even in his stupor. Resistance is also liable to be made by almost all patients just before complete anæsthesia takes place, but the ether rarely requires to be suspended. Occasionally the respiration becomes embarrassed during the period of excitement, partly from the struggle itself, and partly perhaps from the increased flow of saliva, which is a common phenomenon of etherization, or from the position of the tongue or head of the patient, and a condition may sometimes show itself characterized by lividity, rigidity, and convulsive motions of the extremities. These phenomena, it is an observation of Dr. H. J. Bigelow, of this city, are in reality the tetanic symptoms which, as Dr. Brown-Sequard has shown, precede the approach of asphyxia. Although alarming to the inexperienced, the state is in fact devoid of danger, provided 'the ether be momentarily suspended; this being done, the refusal to breathe soon gives place to a long-drawn inspiration, and in most instances complete insensibility immediately ensues. In such a case it is interesting to observe how readily the spasm yields, and how complete is the muscular relaxation which follows the free respiration of air unmixed with ether. It should therefore be borne in mind, that when there is muscular rigidity with lividity, the suspension of etherization will transform this into the relaxation of anæsthesia Persons of intemperate habits succomb to ether slowly, and with greater reluctance and more opposition than persons unused to intoxication.

The pulse should be watched by a competent person from the outset and its failure, either in strength or frequency, lead to a more cautious use of the ether. It must, however, be remembered, that in experiments with anæsthetics upon animals, the heart has been found to be the *ultimum moriens;* the respiratory movements, therefore, should not be forgotten or neglected, but any slowness or irregularity in their performance should at once receive attention. Dr. H. J. Bigelow has drawn attention to the distinction between the effects of anæsthesia upon the pulse of the healthy subject suddenly reduced by accident, and a similar or even stronger pulse in a person exhausted by long and grave disease. In the former case the vitality is unimpaired, and the pulse, even when hardly perceptible, rises with anæsthesia. Ether, therefore, is not to be withheld from a patient to be operated on, even in a state of collapse after severe accident, but great caution is demanded in its use with patients who are near death from chronic and exhausting disease, and who require operations.

The best test of complete etherization is the snoring of the patient; and no operation, unless slight, should be undertaken until this symptom presents itself. The relaxation of the muscles of the extremities may occur without insensibility. The important distinction between *snoring* and *stertor* is, however, to be borne in mind. Whilst the former is caused only by the relaxation of the muscles of the palate, the latter arises from spasm of the vocal cords and partial closure of the rima glottidis, and thus becomes the immediate forerunner of the train of symptoms already referred to as indicative of partial asphyxia. Stertorous respiration demands, therefore, a brief suspension of inhalation; one or two inspirations of fresh air will, as already mentioned, almost instantly dispel the symptom.

Ether may be administered to persons of all ages, from the new-born infant to the octogenarian. There is, however, a condition prone to manifest itself with children, especially those who are weak, strumous or overgrown, which is due to its cumulative properties. It may show itself after almost any degree of etherization, and is characterized by a feeble pulse and slow respiration, not passing off with the readiness usually marking the phenomena of etherization. With young persons a cautious inhalation of five minutes will often induce an anæsthesia of half an hour, an effect wholly out of proportion to that which the same amount of ether would produce in an adult. This state is not a dangerous one, and only requires time to dissipate its symptoms. Compression of the chest will expel the fumes of ether being eliminated from the pulmonary surface, and admit the entrance of a fresh supply of oxygen to stimulate the circulation. The inhalation should therefore be suspended at short intervals with children, and but little either given at a time. Undoubtedly it should also be used cautiously with persons past the middle period of life, of such a general obesity or constitutional condition as may lead to the supposition of a fatty degeneration of the heart. In none of the alleged deaths from ether, however, is there any mention of valvular disease of the heart being found. Of this, then, and of any bad effect upon pulmonary affections, there need be no fear, for we see it constantly administered to persons more or less advanced in phthisis, for the common operation of fistula in ano.

Its subsequent effects are rarely disagreeable. The nausea and vomiting which follow the use of any anæsthetic may be prevented or diminished by giving it upon an empty stomach. Faintness, although a rare event, is occasionally noticed, and demands the ordinary treatment by stimulants. Headache sometimes remains for a few hours, but seldom persists into the following day. We now and then hear of delirium, debility, and the non-return of a full use of the mental faculties, as temporary accidents from the use of ether. Such occurrences must be of extreme rarity, and probably find their explanation as much in the idiosyncrasies of patients as in the effects of the anæsthetic.

The general conclusions which have been arrived at by your Committee may be summed up as follows:—

1st.—The ultimate effects of all anæsthetics show that they are depressing agents. This is indicated both by their symptoms and by the results of experiments. No anæsthetic should therefore be used carelessly, nor can it be administered without risk by an incompetent person.

2d.—It is now widely conceded, both in this country and in Europe, that sulphuric ether is safer than any other anæsthetic, and this conviction is gradually gaining ground.

3d.—Proper precautions being taken, sulphuric ether will produce entire insensibility in all cases, and no anæsthetic requires so few precautions in its use.

4th.—There is no recorded case of death, known to the Committee attributed to sulphuric ether, which cannot be explained on some other ground equally plausible, or in which, if it were possible to repeat the experiment, insensibility could not have been produced and death avoided. This cannot be said of chloroform.

5th.—In view of these facts, the use of ether in armies, to the extent which its bulk will permit, ought to be obligatory, at least in a moral point of view.

6th.—The advantages of chloroform are exclusively those of convenience. Its dangers are not averted by its admixture with sulphuric ether in any proportions. The combination of these two agents cannot be too strongly denounced as a treacherous and dangerous compound. Chloric ether, being a solution of chloroform in alcohol, merits the same condemnation.

R. M. HODGES, S. D. TOWNSEND,
GEO. HAYWARD, C. T. JACKSON,
J. BAXTER UPHAM.

The foregoing report was accepted, and its conclusions adopted by the Society. FRANCIS MINOT, *Secretary*.

Dr. C. T. Jackson, one of the Committee, objects and excepts to the clause in this report in which "all mixtures of ether and chloroform" are denounced, viz: to the words, "the dangers of chloroform are not averted by admixture with sulphuric ether," and to the terms, "treacherous and dangerous compound" of ether and chloroform. He believes that a mixture of four measures of ether and one measure of chloroform may be employed without danger, or with very little danger, and that the risks from chloroform are diminished more than four-fifths by this combination. He believes it to be necessary to have an anæsthetic agent of less bulk than ether, and not so dangerous as chloroform, for army uses, and is satisfied that this mixture, which he has employed and prescribed, completely answers the purpose required.

